# Gastroesophageal reflux disease, Barrett esophagus, and esophageal adenocarcinoma – where do we stand?

**DOI:** 10.3325/cmj.2018.59.97

**Published:** 2018-06

**Authors:** Ivana Mikolašević, Tomislav Bokun, Tajana Filipec Kanižaj

**Affiliations:** 1 Department of Gastroenterology, University Hospital Center Rijeka, University of Rijeka School of Medicine, Rijeka, Croatia *ivana.mikolasevic@gmail.com*; 2 Department of Gastroenterology, Hepatology and Clinical Nutrition, University Hospital Dubrava, University of Zagreb School of Medicine, Zagreb, Croatia; 3 Department of Gastroenterology, University Hospital Merkur, University of Zagreb School of Medicine, Zagreb, Croatia

Almost 25% of all human cancers are located in the gastrointestinal tract (GIT), making it the dominant cancer-affected site. The reason for this could be constant GIT exposure to organ damage and chronic inflammation (1). Despite all medical breakthroughs, less than half of patients survive one year after the esophageal adenocarcinoma (EAC) diagnosis (2). Most malignancies, including most GIT malignancies, are preceded by precursor/premalignant lesions. Premalignant condition for EAC development is Barrett esophagus (BE), a disorder characterized by abnormal transformation of the squamous epithelium. BE is strongly associated with prolonged gastro-esophageal reflux of gastric and bile acids (1,3,4). Since patients with BE carry 30-40 times higher risk for EAC than general population, it is not surprising that in the last 10-20 years the research interest in BE has been growing (3).

The most important risk factor for BE and EAC is gastroesophageal reflux disease (GERD). The majority of GERD patients present with heartburn and effortless regurgitation. GERD is one of the most common indications for visiting primary care physicians and gastroenterologists and the most common GIT-related diagnosis worldwide (1-7). The prevalence of GERD ranges from 8.8% to 25.9% in Europe, 8.7% to 33.1% in the Middle East, 18.1% to 27.8% in North America, 11.6% in Australia, 2.5% to 7.8% in East Asia, and 23.0% in South America (7). Also a significant increase in GERD prevalence has been reported in the last two decades (7). This increase is accompanied by BE prevalence increase, which explains the rapidly increasing EAC incidence in the Western countries (1-7).

Multifactorial pathophysiology and risk factors for GERD are still the subject of many investigations (1,5-7). A recent meta-analysis confirmed age, male sex, tobacco use, and obesity (especially abdominal/central obesity) as the most common risk factors for GERD (3-9). A lot of epidemiological studies have also reported that obesity was an independent risk factor for GERD. In addition, emerging data indicate that obesity, especially central obesity, is strongly associated with complications related to longstanding GERD, such as erosive esophagitis, BE, and EAC (5,10). Epidemic rise in the obesity of adults and children during the last two decades has become a significant health problem in the Western countries, especially since obesity has negative impact on several chronic diseases (11). Increase in obesity incidence is accompanied by an increase in GERD incidence, and since in the next 20 years further increase in obesity incidence is expected, we can anticipate increasingly more cases of GERD as well (11). Obesity changes gastroesophageal anatomy and physiology. People with high body mass index, especially those with high waist circumference, have increased intraabdominal pressure and decreased lower esophageal sphincter tonus. These are often accompanied by hiatal hernia and delayed gastric emptying, which increase the risk of reflux of gastric and bile acids (1,4,5,10,11). Prolonged exposure to gastric acid in GERD causes esophageal mucosa damage. However, in obese people chronic inflammation and metaplastic changes of esophageal mucosa cells are promoted by inflammatory cytokines from the visceral fatty tissue (4,5). Visceral fat is metabolically active and produces a variety of cytokines that promote chronic inflammation and affect esophagogastric motor activity (1,4,5,11). Another factor linking obesity and GERD are hormones, namely increased estrogen activity, which is often observed in obese people. Estrogen stimulates the nitric oxide synthesis, which acts as a vasodilator of smooth muscles, ie, lower esophageal sphincter, which consists of smooth muscles (5,10). Consequently, a major concern for physicians is the increased risk of EAC associated with GERD, the incidence of which is rising in parallel with the rising incidence of obesity. The most challenging issue in the context of GERD, BE, and EAC is screening. Nowadays, the only method for detecting and evaluating GERD complications and BE is upper GI endoscopy accompanied by histology. However, endoscopic screening of all GERD patients is considered to be too expensive, as the disease prevalence is extremely high. Some authors suggest performing endoscopic screening only in high-risk GERD patients, ie, patients with longstanding reflux symptoms or obese patients (4). However, around 40% of EAC patients do not have a history of heartburn, thus surveillance will only target a portion of at-risk patients (4,12). There is also the question whether a single upper GI endoscopic screening is enough or endoscopy should be repeated (1,4,12). Since EAC frequently spreads before the appearance of symptoms (dysphagia), the primary surveillance goal is to detect dysplastic changes and early cancer (4). In addition, there are still no accepted clinical parameters and valid biomarkers for BE and valid methods identifying the patients at higher EAC risk, who require frequent surveillance and early intervention. The only identifying factor and surrogate endpoint for EAC are dysplastic changes of esophageal mucosa. However, a large number of biopsies is required because of sampling errors, and biopsies are characterized by intra- and inter-observer discrepancies in mucosal changes evaluation (1,4).

Having all this in mind, we were delighted to read the article by Markoš et al, published in the current issue of the *Croatian Medical Journal* (13). The authors tried to identify a biological marker of EAC by analyzing the loss of mismatch repair (MMR) system protein expression in metaplasia-dysplasia-adenocarcinoma sequence of BE. This is one of the first studies analyzing microsatellite instability in different stages of BE-EAC sequence. The research demonstrated considerable loss of MLH1 and PMS2 expression in BE-associated carcinoma sequence. However, due to retrospective nature of the study and a small number of patients, it could not be concluded whether these proteins can be used as biomarkers for patient surveillance and treatment. The study shows interesting and potentially important data, warranting further prospective studies with larger number of non-dysplastic BE patients with loss of MMR protein expression. These results are important in the light of increasing obesity incidence and expected increasing incidence of GERD, BE, and EAC. Together with Markoš et al, we can conclude that there is a need for new methods and biomarkers for detection of GERD and BE patients at high risk for EAC. These methods and biomarkers should be highly sensitive, specific, and cost-effective. Risk factors such as male sex and older age cannot be modified, but promotion of primary interventions in terms of healthy life, weight loss, and non-smoking can contribute to GERD prevention (4).
